# News and Coming Events

**Published:** 1907-10-19

**Authors:** 


### NEWS AND COMING EVENTS.

A CONVERSAZIONE will be held on Wednesday, October 23 next, at 9 P.M., at St. Bartholomew's Hospital. The new out-patient department will be open to inspection.

THROUGH the liberality of Miss Standering, of Selby, a memorial ward in connection with the Selby Cottage Hospital has been erected at a cost of over £700, and endowed with £3,000.

THE Right Hon. the Lord Mayor of London (Sir Wm. Purdie Treloar Bart.), will be present as the guest of the evening at the fifth annual dinner of the Hospital Officers' Association, which will take place on November 2 at the Gaiety Restaurant.

LORD CAWDOR will take the chair at a festival dinner at the Hôtel Ritz on Wednesday, November 20 next, in aid of the Building Extension Fund of the London Homœopathic Hospital, Great Ormond Street. It is estimated that £30,000 will be required to extend the hospital on its own freehold site, and a balance of £13,250 is still required to complete the £30,000.

IN the Guntur District (Madras Presidency) leprosy is very prevalent, and in order to provide a home for these destitute outcasts, the Mission to Lepers has decided on the erection of an asylum at Bapatla. Already there are upwards of 100 lepers of all ages and various castes sheltered in temporary houses. The Society needs £400 to £500 to provide comfortable houses, dispensary, etc., for 150 inmates. Regular medical treatment will be afforded them, and experience has shown in the other fifty asylums of the Mission that great alleviation of their sufferings is possible though actual cures cannot be reported.

THE forthcoming bazaar to be held at the Camberwell Baths in aid of the fund for the removal of King's College Hospital to South London bids fair to become a very large and popular undertaking. It will be opened by their Royal Highnesses the Duke and Duchess of Connaught on the first day, October 29. It has already been called "The Bazaar of all Creeds and Politics," owing to the course adopted by the Mayor of Camberwell in inducing people of every grade of religious and political thought to take part in it. Fancy dress will be worn, but the costumes will not be confined to one period, as in the case of the Elizabethan Fair and Fête held last year.

FOR twenty-five years Sir James Reckitt, Bart., has been a member of the Board of Management of the Royal Infirmary, Hull, and for seven years he has held the position of President of the Board. He has been a staunch friend to the institution, which has benefited largely by his generosity. As a small token of their appreciation of his work on behalf of the infirmary his colleagues on the Board, the medical staff of the infirmary, and the Working-men's Committee recently presented him with his portrait. The portrait is the work of Mr. J. H. Bacon, and is a striking likeness of the President of the Board. The presentation was made by Mr. C. D. Holmes, J.P., at the last meeting of the Board.

### THE LONDON MEDICAL EXHIBITION.

THE Editor and staff of the *British and Colonial Druggist* are to be congratulated on the success of the London Medical Exhibition, which was closed on Friday last. For four days the Royal Horticultural Hall, Vincent Square, where the Exhibition was held, was well visited by medical men and their friends, and next year's exhibition will be looked forward to with interest by those who had the pleasure of inspecting the exhibits. We have already given a short notice of the main features of the various stalls, but a few remain for more extended mention. The Hospitals and General Contracts Co. had on view a fine show of their aseptic furniture. The Equipoise specialities, in the shape of beds, mattresses, and invalid furniture attracted a good deal of attention. The special mattress exhibited by this firm is claimed to be the only "self-adjusting mattress made," and can be placed and used on any bedstead. Its position and angle of inclination may be altered by the occupant of the bed himself without exertion, and held by means of a special catch. This is no mere mechanical action; the mattress is directly under the control of the occupant, who alters the position with the greatest ease. The same arrangement has been adopted in the adjustable "Equipoise" couch, which the company has had the honour of supplying by special command to her Majesty the Queen for use at Buckingham Palace. Special consulting-room couches are made by this firm, and appear to answer their purpose excellently. The stall of Messrs. Thos. Christy and Co. held many interesting therapeutical novelties. There could be seen "Adnephrin" and "Alphogen" (Frederick Stearne and Co.), the latter one of the most powerful non-toxic germicides on the market; "Anasaroin," an American preparation now used with great success in cases of dropsy; "Cocaine Discoids," specially prepared for dental work; "Lysoform," an ideal antiseptic containing a high percentage of formaldehyde, and specially suitable for surgeons' use; Pertussin, a preparation which has been used with striking benefit in cases of whooping cough, together with old and well-known favourites such as Gonosan and Glyco-thymolin. Meister, Lucius and Brüning showed specimens of their pure therapeutic preparations, such as Albargin, a gelatose silver compound, neutral in reaction, and entirely soluble in water, and a powerful germicide and antiseptic; Pyramidon, both in the form of salicylate and bicamphorate, a powerful antipyretic and antineuralgic; Suprarenin, a synthetically prepared hæmostatic and astringent. Messrs. C. J. Hewlett and Son showed many of their special preparations. Their Elixir Aceto-Salicylic acid is perfectly soluble, permanent and palatable, a valuable antipyretic and analgesic, specially recommended for gout, articular rheumatism, and neuralgia. Hæmorrhaline is a preparation containing acetate of lead, witch hazel, and morphine, which will be found serviceable in the treatment of piles. Among their other preparations, too numerous to specify individually are Lin. Betulæ Co., Mist. Daminæ Co., Mist. Nucis Vom. c. Rheo Co., and the excellent Mist. Pepsinæ Co. c. Bismutho. A word, too, must be said of their Mist. Neuro Co., a mixture containing quinine, nux vomica, caffeine, phenazone, gelsemium, and potassium bromide, which has won high appreciation from the profession in the treatment of obstinate cases of neuralgia. E. Merck's preparations, as usual, came in for a good deal of attention. This firm showed samples of their Fibrolysin, which has lately been used in the treatment of gastric adhesions; Tropacocaine Hydrochlorate, which has been used as a local anæsthetic in major surgical operations with striking success; Dionine, Stypticine, and Veronal. Messrs. Hearon, Squire and Francis exhibited a remarkable series of opium preparations, including samples of English opium from Lincolnshire. The Miol Manufacturing Co. had an attractive and interesting stall, at which specimens of their Miol and Miotannol were on view.

